# In Vitro Antifungal Activity of Thiosemicarbazide Derivatives with a Nitroimidazole Moiety Against *Trichophyton* spp. Dermatophytes

**DOI:** 10.3390/molecules30224439

**Published:** 2025-11-17

**Authors:** Sylwia Andrzejczuk, Urszula Kosikowska, Monika Wujec

**Affiliations:** 1 Department of Pharmaceutical Microbiology, Medical University of Lublin, Chodzki Str. 1, 20-093 Lublin, Poland; 2 Department of Organic Chemistry, Faculty of Pharmacy, Medical University of Lublin, Chodzki Str. 4A, 20-093 Lublin, Poland; monika.wujec@umlub.edu.pl

**Keywords:** *Trichophyton* spp. dermatophytes, antidermatophytic activity, nitroimidazole moiety, thiosemicarbazide derivatives

## Abstract

Dermatophytes can cause infections of the skin, hair and nails. This study aims to investigate the thiosemicarbazides with nitroimidazole moiety against *Trichophyton* spp. The activity of fourteen thiosemicarbazide derivatives was evaluated against *Trichophyton* spp. The minimal inhibitory concentration (MIC) and minimal fungicidal concentration (MFC) showing 50% and 90% reduction in fungal growth after 4–7 days of incubation (MFC_50_ and MFC_90_) were used. The **6** and **11** (MICs ≤ 125 µg/mL), followed by the **3**, **5** and **7** containing a fluorophenyl group (MIC = 125 µg/mL, MFC = 125–250 µg/mL) exhibited the best activity and specifically *T. mentagrophytes*, respectively. Fluorine-containing derivatives (**5**–**9**) demonstrated 2–4-fold higher activity (MIC *=* 31.25–1000 µg/mL) against *T. rubrum* than *T. mentagrophytes*, than their chlorinated counterparts (**2**–**4**) with MIC = 62.5–500 µg/mL. The position of the fluorine atom within the phenyl ring was important, as observed for derivatives with fluorine in the *meta* position (**3**, **6**), while the *para* position was associated with enhanced selectivity. A methoxy group in the *meta* position of the phenyl ring exhibited the strongest, broadest-spectrum activity. Notably, the introduction of the trifluoromethylphenyl moiety (pharmacophore) led to the disappearance of antifungal properties.

## 1. Introduction

Dermatophytes are fungi that can cause dermatophytosis due to invade the keratin layer and infecting human skin, hair, and nails. These fungi’s secondary metabolites may play a role in the immune system regulation or in controlling secondary infections with other pathogens. The predominant challenges are erroneous laboratory diagnosis and the inability to differentiate dermatophyte species. *Trichophyton rubrum* has been identified as one of the most prevalent dermatophyte species isolated as a primary cause of skin and nail infections in adults in studies conducted by Polish research centers. *T. rubrum* has been documented to account for over 70% of reported cases of dermatophytosis, followed by *T. tonsurans* [1,2] and *T. mentagrophytes*/*interdigitale* complex [3]. The results from Poland are in accordance with global observations, with *T. rubrum* maintaining its status as a predominant causative agent of tinea pedis/unguium on a global scale (as evidenced by numerous cohorts) [3,4,5,6], with a prevalence ranging from 20 to 25% [5,6]. *Trichophyton indotineae*, which is rapidly spreading beyond South Asia, is proving to be a new challenge [7]. Furthermore, a multitude of isolates resistant to terbinafine have been documented [7]. The global propagation of antifungal-resistant *T. indotineae* (formerly designated *T. mentagrophytes* genotype VIII) represents a significant public health concern in the contemporary era, especially within the context of southern Asia, yet it has now been identified across the globe, including in Europe, North America, Australia, and the UK [7,8]. This fungus is responsible for initially the groin (tinea cruris), widespread, persistent skin infections affecting multiple body sites that are frequently resistant to standard first-line treatments such as terbinafine. Resistance to this fungus has been linked to the inappropriate use of topical antifungal and corticosteroid combinations [9,10,11].

The secretion of lytic enzymes and systems for capturing released nutrients plays a pivotal role in the pathogenesis of dermatophytosis. These mechanisms are critical for the ability of dermatophyte fungi to utilize human and animal tissues as a source of nutrition. Furthermore, dermatophytes fulfil all four criteria necessary for the invasion of a healthy human organism, i.e., growth at body temperature, the ability to bypass or penetrate surface barriers, the capacity to lyse and absorb tissue, and resistance to the immune system’s defense mechanisms, including elevated body temperature [12].

Factors influencing the high prevalence of dermatophytoses/-mycoses in connection with the relation to the interaction member and the environment are also: a broad spectrum of secreted exoenzymes, regional occurrence of specific species, ability to master various ecological niches. Several additional factors have been identified as being associated with patient characteristics that significantly impact the incidence of dermatophyte infections. These factors include, e.g., age, gender, damage or maceration of the epidermis, genetic susceptibility to disease, mechanical damage to the skin, local reduction in the immune barrier associated with circulatory disorders caused by compression, possible contact with dermatophytes, and exposure to a large number of spores due to socioeconomic or occupational status, and the geographical location of residence [5,9,12,13].

In clinical practice, a broad spectrum of antifungal medications with different mechanisms of action is available. The utilization of antifungal agents is associated with a significant risk of adverse effects, thereby complicating the management of superficial dermatophyte infections in specific demographic groups, including the elderly, or childhood, metabolic disorders, immunocompetency, and pregnancy. A salient feature of substances employed in the treatment of superficial fungal infections is their capacity to readily permeate the stratum corneum while maintaining a consistent concentration over the duration of the treatment period [12,14]. Furthermore, there is currently no vaccine available against dermatophyte infections in humans [14,15], which remains an important element of prevention and a potential first step in the implementation of therapies [15].

The limited number of cellular targets and the severe, difficult-to-avoid side effects of the antifungal agents that are currently available are other issues that give rise to many concerns. Despite the existence of a number of groups of antifungal drugs available for clinical use in dermatophytosis, these drugs only act on a limited number of cellular targets, including the cell membrane, cell wall, nucleic acid synthesis, or fungal growth and cell division [14]. Consequently, dermatophytes may exhibit increasing tolerance or resistance to these agents, irrespective of the method and timing of administration. Furthermore, the mechanisms of action of commonly used antifungal medicines overlap, and this may contribute to the development of multidrug-resistant phenotypes observed in at least several pathogenic fungi [11,12,14].

The mounting resistance exhibited by dermatophytes to clinically and veterinary available antifungal medications, in conjunction with the escalating prevalence of complex infections of this etiology in both humans and animals, underscores the imperative for augmented research endeavors and the ongoing refinement and formulation of novel, alternative public health strategies. The identification of novel pharmaceutical agents for utilization in antifungal therapy or prophylaxis constitutes a considerable challenge, for a number of reasons. Firstly, it is important to note that the majority of cutaneous fungal infections affect individuals with compromised immune systems, frequently in conjunction with chronic and/or metabolic diseases. Consequently, these patients are more dependent on the efficacy of antifungal medications than those with normal immunity. Secondly, the development of effective and safe antifungal medications is challenging due to the similarity of numerous fundamental biological processes between fungi and humans. Consequently, it is challenging to identify chemical compounds that inhibit the growth of the pathogenic fungi without adversely affecting the host organism. Notwithstanding the numerous challenges encountered, avenues for the development of innovative therapeutic interventions do exist. Recent advancements in the field of mycology, including the understanding of the fungal life cycle, the study of functional genomics, proteomics, and gene mapping, have created new possibilities for the identification of novel cellular targets for antifungal agents, thereby expanding the current antifungal alternatives [4,5,6,7,10,13,16].

Presently, synthetic compounds exhibiting particular anti-fungal properties against dermatophytes are the focus of scientific research and preclinical and/or clinical trials. These include as follows: sertaconazole (which exhibits enhanced drug delivery and retention), eberconazole (which demonstrates higher retention in the stratum corneum and increased efficacy), and well-described terbinafine (which presents enhanced drug delivery, and its potent fungicidal properties, particularly against *T. tonsurans*), ciclopirox 8% lacquer (demonstrating water-soluble film, increased compliance, and better cell penetration), econazole nitrate 1% foam (which exhibit better mycological cure, and easier application method), as well as luliconazole (active in reducing fungal colony-forming units) [13].

Dermatophytoses are considered to be prevalent superficial infections that occur worldwide in both humans and animals. The principal challenges in diagnosing and treating these infections for clinicians and researchers are: the increasing frequency and recent advances in the identification of dermatophytes (fungi that typically infect the skin or hair); the prolonged duration of treatment; the limited number of available antifungal agents; the numerous side effects of existing drugs; and finally, the emergence of antifungal resistance. The endeavor to identify and synthesize novel antifungal medications is hindered by the striking similarity between the structural configurations of human and fungal cells. Moreover, establishing treatment standards necessitates the acquisition of the results of either in vitro or in vivo studies of clinically utilized antifungal substances, alongside the assessment of the reliability of developing an efficacious vaccine. The identification of dermatophyte species is complicated, and there is a need to correctly determine whether the infection is of zoophilic origin. These issues make prophylactic and therapeutic problems worse.

Furthermore, in order to ensure optimal treatment, it is imperative to incorporate in vitro and in vivo evaluation of novel antifungal compounds, particularly those of natural provenance, alongside the exploration of their chemical substitutes [13,17,18].

This study aims to investigate the structure/activity relationship of the thiosemicarbazide derivatives with nitroimidazole moiety against *Trichophyton* spp. strains, either reference or clinical isolates. All these compounds were chemically characterized earlier [19,20] including antibacterial properties, but without anti-dermatophytes activity examination.

## 2. Results

As demonstrated in Table 1, the anti-dermatophyte activity analyses indicated that **6**, **11**, exhibited the most potent activity against the majority of the dermatophyte strains among all fourteen tested compounds (with MIC ≤ 125 µg/mL). This finding suggests that both compounds demonstrate robust and broad-spectrum activity against the *Arthrodermataceae* family. It is noteworthy that both thiosemicarbazide derivatives contain substituents in the *meta* position of the phenyl rings (**6**—fluorine; **11**—methoxy).

**Figure A1:**
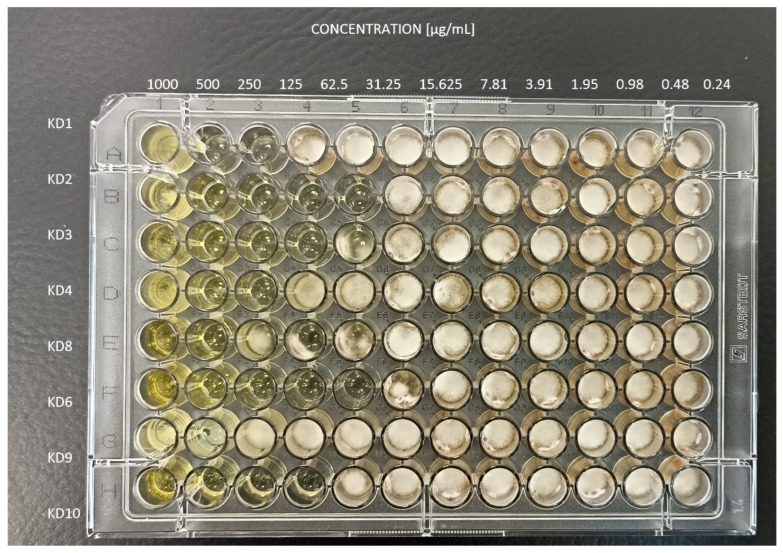
Microtiter plates after 4 days of incubation at 25 ± 2 °C of *Trichophyton rubrum* clinical isolate.

Both derivatives exhibited an inhibition of the formation of hyphae and micro- and macroconidia of dermatophytes, with the degree of inhibition being dependent on the concentration of the compound (Figure A1). The effect was sustained for a period of between four and seven days during the incubation phase, which was conducted at a room temperature that was maintained at a high level of humidity (Figure A1).

The second group of compounds, which presented efficacy against *T. mentagrophytes* strains tested, included **3**, **5**, and **7**, with MIC values of 125 µg/mL and MFC_50_ and MFC_90_ ranging from 125 to 250 µg/mL. It was established that each of the compounds was fungicidal to *T. mentagrophytes*. Two of these compounds were found to contain a fluorophenyl group within their structure.

A third group comprised **2**, **3**, and **5** compounds, which demonstrated notable selective activity against both *T. rubrum* strains tested. It was observed that all derivatives manifested the lowest MICs and MFCs_50_ = 62.5 µg/mL and MFCs_90_ = 125 µg/mL for *T. rubrum* ATCC 28188. In the case of *T. rubrum*, a clinical isolate of human origin skin infection, MFCs_50_ and MFCs_90_ exhibited two-fold higher values. In addition, **7** demonstrated a noteworthy, good anti-dermatophytic and fungicidal properties, directed at *T. rubrum* (MIC = 31.25 and 62.5 µg/mL for reference strain and clinical isolate, respectively). Evidence was presented that indicated the presence of a chlorine atom in position *ortho* and *meta* on the phenyl ring of **2** and **3**, respectively, and a fluorine atom in *ortho* and *para* positions of the phenyl ring of **5** and **7**, respectively.

**Figure 1:**
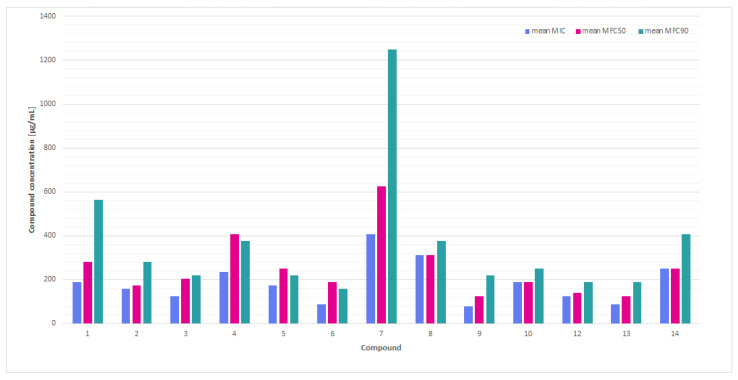
A comparison of the mean minimum inhibitory concentrations (MICs) and minimum fungicidal concentrations (MFC_50_ and MFC_90_) values for thiosemicarbazide derivatives with nitroimidazole moiety against the tested *Trichophyton* spp. dermatophytes.

During the analysis of the present study results, potentially fungistatic, non-fungicidal compounds (with low MIC but high MFC values, Figure 1) were also identified: **2** and **3** exhibited relatively low MICs, yet MFC_90_ frequently exceeded 500 µg/mL, a threshold also met by **6**, **11,** and **3** (Table 1). These compounds, distinguished by their low MICs and moderate MFCs_90_, merit consideration as potential candidates for fungicide applications. It is hypothesized that these thiosemicarbazide derivatives with nitroimidazole moiety could be a multifaceted pharmaceutical agent that finds application in a variety of preparations intended to provide support in cases of skin or mucous membrane infections. It could also be employed in the synthesis of disinfectants.

A comparison of the average MIC, MFC_50_, and MFC_90_ values (Figure 1) showed that compounds such as **3**, **6**, **11**, and exhibit low MIC and relatively low MFC_90_, making them potentially fungicidal and effective against the tested dermatophytes. The compounds **9** and **14** exhibited the highest values for both parameters, indicating their minimal effectiveness.

**Figure 2:**
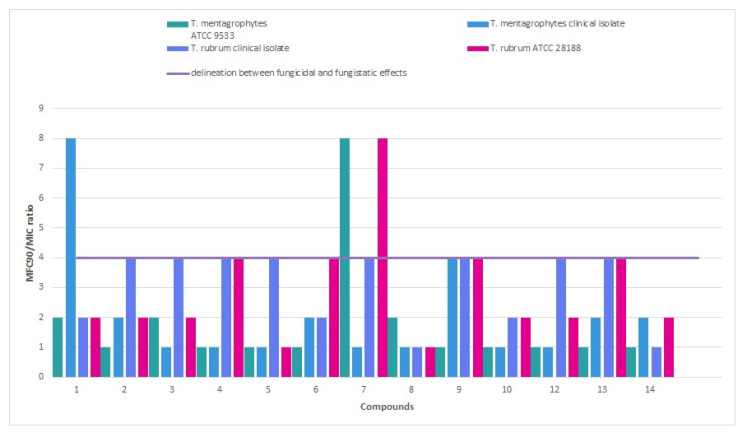
Fungicidal and fungistatic delineation among thiosemicarbazide derivatives with nitroimidazole moiety based on MFC_90_/MIC ratio.

The calculation of the fungicidal index (MFC_90_/MIC) yielded values approximating 1.0 (e.g., **6**, **11**), suggesting fungicidal activity, as opposed to values exceeding 2.5 and more (e.g., **2**, **3**), which indicate fungistatic activity (Figure 2). It has been revealed that the following compounds **6** and **11** were the most promising with *meta*-fluoro (**6**) and *meta*-methoxy (**11**) substituents on the phenyl ring and position relation to antidermatophytic activity.

**Figure 3:**
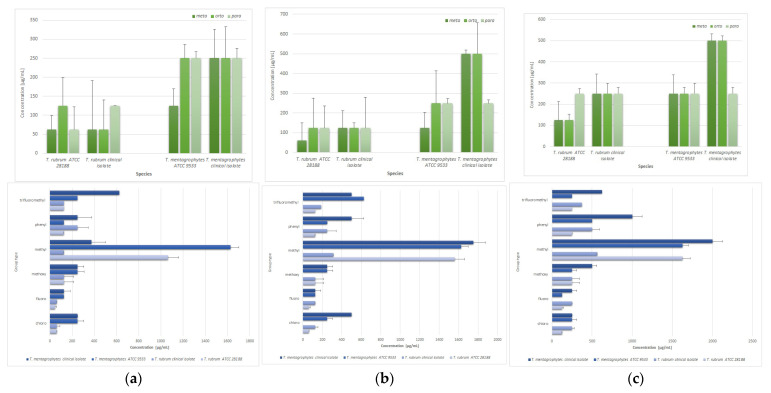
The effect of substituents of thiosemicarbazide compounds on the mean microbiological parameters: (**a**) MIC, (**b**) MFC_50_, and (**c**) MFC_90_ in relation to different species of the genus *Trichophyton*; standard deviation calculated from three replicates.

The investigation sought to ascertain whether a correlation exists between the chemical structure of thiosemicarbazide derivatives and their capacity to exhibit antimicrobial activity against *Trichophyton* spp. Specifically, the study examined the influence of substituent type and position on the derivatives’ activity (Figure 3). The results demonstrated that, in general, compounds with substituents in *meta* position on the phenyl ring consistently exhibited high efficacy, as evidenced by the lowest MIC and MFC values recorded for both *T. rubrum* ATCC 28188 (mean MIC and MFC_50_ = 62.5 µg/mL, mean MFC_90_ = 125 µg/mL) and *T. mentagrophytes* ATCC 9533 (mean MIC and MFC_50_ = 125 µg/mL, mean MFC_90_ = 250 µg/mL). The compound with *meta*-fluorophenyl substituent (**6**) demonstrated the most effective MIC values of 31.25 µg/mL, while the compound with chlorophenyl substituent in the same position (**3**) exhibited the most effective MFC_50_ and MFC_90_ of 62.5 and 125.0 µg/mL values for *T. rubrum* ATCC 28188, respectively. In turn, for *T. mentagrophytes*, the compound with the chlorine in the *meta* position on the phenyl ring (**3**) yielded the optimal MIC and MFC_50_ results of 125.0 µg/mL, respectively, while the compound with the *meta-meta*-fluorophenyl substituent (**6**) exhibited the most effective MFC_90_ result of 125.0 µg/mL. Conversely, the combination exhibiting the lowest efficacy was the compound with *meta*-methyl substituent, which consistently demonstrated the worst (highest) MIC values of 2000 µg/mL for both *Trichophyton* spp. reference strains. This finding is indicative of minimal biological activity. No statistically significant differences were found based on the current dataset.

## 3. Discussion

Thiosemicarbazides are chemical compounds that have been the subject of scientific interest for centuries [21,22,23,24,25,26]. This is primarily due to their promising diverse biological properties, which play a significant role in organic chemistry and medicine. The observed effect can be attributed to the thiosemicarbazide group (-NH-CS-NH-NH_2_), which has been documented to possess a wide range of antimicrobial properties, comprising the activity of antibacterial (particularly against Gram-positive bacteria, including staphylococci, and enterococci) [20,21,27], anticancer [19,20], antifungal [21,22,28,29], and antiviral [19,20]. It has been demonstrated that thiosemicarbazides exhibit antibacterial properties, thus rendering them promising candidates for the development of alternative therapeutic options with efficacy similar to that of antibiotics and antimycotics [9,21,26,28] With regard to chemical modifiability, they may exhibit a distinct advantage over other synthetic compounds.

It is hypothesized that these compounds may act through various mechanisms involving both DNA gyrase and topoisomerase IV inhibition, which are found exclusively in bacteria [21,22,23,24,25]. The presence of these compounds in the microbial environment has been demonstrated to result in the disruption of DNA replication and bacterial cell death. Furthermore, the antifungal properties of thiocarbazides are primarily ascribed to their ability to disrupt the function of fungal cell membranes and inhibit protein synthesis, ultimately resulting in fungal cell death. A plethora of studies on various compounds have demonstrated their effectiveness in combating various fungal pathogens, such as *Aspergillus flavus* or *A. parasiticus* mold fungi [28]. This finding indicates that these compounds may have potential practical influence or application in the treatment or prophylaxis of fungal infections.

Clinicians, in conjunction with mycologists have identified the pressing necessity to enhance awareness among healthcare professionals and the general public, to facilitate enhanced laboratory detection, expedited treatment, and effective measures for infection prevention and control [30]. Research conducted to date has documented their antifungal potential, particularly about dermatophytes of the genus *Trichophyton*. It has been demonstrated through rigorous research that the incorporation of a nitroimidazole moiety and a thiosemicarbazide structure within a compound can result in the disruption of fungal growth and cell wall integrity. Dincel et al. [26] synthesized new hydrazinecarbothioamide, 1,2,4-triazole, and 1,3,4-thiadiazole derivatives, which demonstrated antifungal activity against *Trichophyton* spp. strains (e.g., *T. mentagrophytes* var. *erinacei* NCPF 375 and *T. tonsurans* NCPF 245). The authors of the study concluded that hydrazinecarbothioamide derivatives were predominantly responsible for the MIC of 64 μg/mL and above for *T. mentagrophytes*. Concurrently, triazole and thiadiazole derivatives exhibited enhanced activity in comparison to the aforementioned compounds. Similarly, the antifungal activity of novel 1,2,4-triazolylmercaptoacetylthiosemicarbazide and 1,2,4-triazolylmercaptomethyl-1,3,4-thiadiazole analogs were investigated against *T. mentagrophytes* and *T. rubrum* isolates [25]. It was established that a number of compounds which were subjected to testing demonstrated the capacity to exhibit anti-dermatophytic properties, with MIC values ranging from 4 to 8 μg/mL. Recent studies have identified two new compounds, 2-(4-methylphenyl)-3-((6-phenylimidazo [2,1-b]thiazol-3-yl)-acetamido)-4-thiazolidinone and 2-(4-chlorophenyl)-3-((6-phenylimidazo [2,1-b]thiazol-3-yl)-acetamido)-4-thiazolidinone, as the most active against *T. mentagrophytes* var. *erinacei* NCPF-375, with an MIC of 3 μg/mL [22]. Moreover, the findings of that study demonstrated that trifluoromethylthiolated derivatives of cinnamate and chloroaromatic motives exhibited the most effective inhibition profile (MICs = 2.08–50 μg/mL and MFCs = 3.12–50 μg/mL) against *T. mentagrophytes* TME16 and *T. rubrum* TRU45 [23]. Almost every research study mentioned omitted any reference to the MFC/MIC ratio. Consequently, it was not possible to ascertain whether the activity was fungistatic or fungicidal. The findings of the aforementioned studies do not permit the establishment of a definitive relationship between structure (i.e., aliphatic or aromatic substituents) and activity. However, it can be hypothesized that these compounds have potential for further investigation in the development of new antifungal drug candidates.

Modifications of thiosemicarbazides can be implemented with ease in order to enhance their properties, and to facilitate the synthesis of novel compounds that have the potential to be therapeutic. Moreover, these modifications also have the potential to enhance the stability and consistency of the substances. The toxicity of these compounds is known to vary depending on the specific application and the chemical structure of the substance in question [14,21,26]. As a result of this study, it was found that the introduction of a halogen atom has a pronounced impact on biological activity. Fluorine-containing compounds (**5**–**9**) exhibit higher activity than their chlorinated analogues (**2**–**4**). The position of the fluorine atom on the phenyl ring plays a decisive role: the highest activity was observed for the derivative bearing fluorine in the meta position (**6**). In contrast, substitution at the position para was associated with increased selectivity of action. Among electron-donating substituents, only the derivative carrying a methoxy group in the meta position on the phenyl ring displayed strong and broad-spectrum activity, whereas relocation of this substituent to the ortho or *para* position resulted in a complete loss of activity. Notably, the introduction of the trifluoromethylphenyl moiety, a commonly recognized pharmacophore, led to the disappearance of antifungal properties.

It is noteworthy that the *meta* position of substituents in the phenyl ring was of critical importance for achieving effectiveness among all compounds that were tested. The present study hypothesises that the *meta* position may be a critical factor influencing effectiveness, as, depending on the type of substituent, it is associated with both the highest and lowest antifungal effectiveness values. The investigation revealed that compounds with chlorophenyl and fluorophenyl substituents exhibited high activity against dermatophytes, and especially if these atoms were in the *meta* position on the phenyl ring, be consistently associated with the highest efficacy (lowest MIC and MFC values) for both *T. rubrum* ATCC 28188 and *T. mentagrophytes* ATCC 9533. This finding may indicate that these combinations could be promising for the development of antifungal compounds specific to dermatophytes of the genus *Trichophyton*. Despite the evident variations in mean microbial activity across diverse substrate categories, statistical analysis failed to demonstrate statistical significance for *T. rubrum* ATCC 28188. This finding necessitates further research to ascertain whether these variations are attributable to chance or whether there exists a robust, consistent correlation between these particular structural characteristics and microbiological activity.

The derivative with the substituents with a chlorine atom demonstrated the highest level of activity against all fungal species, including the *T. rubrum* clinical isolate [22]. The investigation revealed that the incorporation of two electronegative chlorine atoms as substituents resulted in a substantial augmentation of the antimicrobial activity of 1,2,4-triazole derivatives. It was hypothesized that an increase in the volume of the group or aromatic group in the phenyl ring would result in a decrease in biological activity. Yamaguchi et al. [29] introduced the thiosemicarbazide into the known structure of camphene. It has been demonstrated that this results in increased antifungal activity, as evidenced by a decrease in MIC values from 548 to 55 μmol/L and an increase in MFC from 110 to >735 μmol/L. This finding provides a foundation for hypotheses regarding its significant effect on the cell walls of *T. mentagrophytes* ATCC 11481 and dividing cross walls (excretions of fibrillar materials and swollen hyphae). The hypothesis that this compound may affect the structure of the fungal cell wall and damage it, or may interfere with its formation during cell division, growth, and morphogenesis of dermatophytes, has been postulated [29]. This approach to the synthesis of new derivatives has the potential to yield interesting compounds with greater biological activity in pharmacological studies. In the course of their research, Kaplancıklı et al. [27] discovered that the reaction of 4-[4-(trifluoromethyl)phenyl]thiosemicarbazide with aromatic aldehydes resulted in the synthesis of fluorosubstituted derivatives. These derivatives were found to demonstrate a wide-ranging spectrum of activity against a variety of microbes, including mycobacteria (*Mycobacterium tuberculosis* H37Rv), enterococci (*Enterococcus faecalis* ATCC 29212), staphylococci (*Staphylococcus aureus*), and yeasts (*Candida glabrata*). The MIC values of these derivatives ranged from 200 to 1600 µg/mL, indicating their potential for use in medical treatment. It was determined that the incorporation of fluorine into a compound’s chemical structure can enhance the acidity of numerous compounds, thereby increase their lipophilicity and facilitating their permeation through biological membranes [30]. A similar type of action was determined for another halogen, i.e., bromine substitution on the aromatic ring. It was observed that this modification has the capacity to enhance antibacterial activity by exerting influence on electronic properties, increasing polarization, improving hydrophobic contacts in the binding site, and the ability to interact with microbial targets such as bacterial enzymes [26]. It is well established that halogens (e.g., chlorine or iodine) are potent disinfectants. They are capable of eradicating microbes by inducing oxidative stress, thereby destroying their essential cellular components and inhibiting vital processes. These substances are powerful oxidizing agents that cause indiscriminate damage to bacterial cell walls, membranes, nucleic acids, and cellular proteins. In addition, they disrupt oxidative phosphorylation, a process that is critical for bacterial cell survival. While chlorine is generally more effective than the iodine in inactivating vegetative bacteria, it is less potent against bacterial spores [31]. Conversely, fungitoxicity can be impacted by substitutions, such as those involving mono- and dichloroquinolinols in different positions [32]. It is believed that halogens can also inhibit the growth and multiplication of dermatophytes by enhancing antifungals, such as polyenes (e.g., amphotericin B), through membrane destabilization [22,23]. Such molecules are typically more lipophilic and, consequently, more lipid-soluble. This lipophilicity facilitates the integration of fluorinated molecules into membranes, resulting in a substantial enhancement in permeability [23]. As demonstrated by the presence of high concentrations of the simple fluoride ion, the production of broad fungicidal compounds that contain fluorine, such as fluoroquinolones and fluorinated flavonoids, can also be expected to show antifungal activity. Such compounds are capable of affecting the fungal cell wall or of acting through other mechanisms. Fluconazole, an antifungal agent containing a fluorine atom, is effective in the treatment of fungal infections by accumulating in the outermost layers of the human or animal skin. However, the distribution of flukonazole within the stratum corneum and sebaceous glands is a complex process. The fluoride ion has been demonstrated to inhibit fungal growth by blocking the functions of essential metabolic enzymes [33].

While the findings of this study do not attain statistical significance, they may imply a correlation between the nature and position of the substituents on the phenyl ring (3-chlorophenyl and 3-fluorophenyl), with reduced MIC values, indicating heightened antifungal activity. Conversely, the methyl substituent and the *meta* position have been linked to elevated MIC values (indicating reduced activity). The observed patterns may serve as hypotheses for future studies using larger data sets, including a greater number of isolates from clinical materials, or more targeted experiments to confirm possible correlations.

It is acknowledged that the data obtained is subject to certain limitations. The absence of statistical significance may have been primarily influenced by the limited sample size in each category, both in terms of microbiological parameters, including the number of dermatophyte isolates, and physicochemical parameters (number of compounds with identical chemical structures). This may have resulted in a reduction in the power of statistical tests in detecting real differences within the studied derivatives.

## 4. Materials and Methods

### 4.1. Synthetic Compounds

All fourteen thiosemicarbazide derivatives with nitroimidazole moiety were described previously [19,20] Their structural formulas are revealed in Table 2.

### 4.2. Microorganisms

Two clinical isolates of dermatophytes (from human skin infections) comprised *Trichophyton rubrum* and *T. mentagrophytes*. The reference strains of *T. rubrum* ATCC 28188 and *T. mentagrophytes* ATCC 9533 were obtained from the American Type Culture Collection. The collection of all dermatophytes was conducted at the museum of the Department of Pharmaceutical Microbiology, Medical University of Lublin, Poland. The isolates were stored frozen in the Sabouraud broth medium (Biomaxima, Lublin, Poland) with 30% (*v*/*v*) of sterile glycerol at a temperature of −70 °C. Freshly thawed strains were utilized for the analyses, which were conducted on Sabouraud agar medium with the incorporation of chloramphenicol (Biomaxima, Lublin, Poland) for a duration of five to seven days at 25 ± 2°C temperature in a humid chamber.

### 4.3. Preparation of Trichophyton *spp.* Inoculum

Suspensions of the tested dermatophytes were prepared according to the EUCAST [34,35]. For this purpose, using a moistened sterile swab, microconidia were collected in a sterile tube containing 5 mL of sterile distilled water, the suspension was centrifuged for 15 s, and then transferred to a sterile syringe with a pore diameter of 11 µm filter, filtered, and collected in a sterile tube. The reconstituted suspension was adjusted to a McFarland density of 0.5 (5 × 10^5^ CFU/mL) in sterile 0.85% NaCl. The suspensions of the tested microorganisms obtained in this way were diluted in a 1:10 ratio in the sterile Mueller-Hinton Broth with 5% of glucose (Biomaxima, Lublin, Poland), to obtain a final density corresponding to 5 × 10^6^ CFU/mL, and added to all the wells of the plate in a volume of 5 µL using an automatic pipette.

### 4.4. Minimal Inhibitory Concentration (MIC) Assay

The studied compounds were screened for antifungal activities using a microdilution broth method according to the protocols of the European Committee on Antimicrobial Susceptibility Testing (EUCAST) on Mueller-Hinton Broth with 5% of glucose [33].

Sterile 96-well polystyrene microtiter plates (Medlab, Raszyn, Poland) were prepared by adding 200 µL of the tested compounds diluted in the appropriate broth medium (with an initial concentration of 2000 µg/mL). Then, 100 µL of sterile medium was added to each well. To obtain final concentrations of the tested compounds in the range of 0.98 to 2000 µg/mL, serial twofold dilutions were performed. One hundred microliters of the diluted compounds from the first wells (2000 µg/mL) were taken and diluted. After 4-day incubation at 25 ± 3 °C, the MICs were determined visually, as a result of an optically clear well, and at 600 nm by using a spectrophotometer microplate reader ELx800 (Biokom, Janki, Poland). Concurrently, microscopic observations were conducted after 4 and 7-day incubation using the Olympus DP22 automated inverted light microscope, CellSens Dimensions 2.3 software (2000× magnification, 20 µm scale bar; Olympus Corporation, Hachioji-shi, Japan).

An appropriate positive (containing the inoculum of all microbial strains with fluconazole; Glentham Life Sciences, Wiltshire, UK) and negative controls (containing the tested extracts without the inoculum, including a sterile broth medium) were included on each microplate.

### 4.5. Minimal Fungicidal Concentration (MFC) Assay

The minimal fungicidal concentration (MFC) was visually recorded as the lowest concentration that establishes a predetermined reduction of 50% (MFC_50_) and 90% (MFC_90_) of fungal growth after 5–7 days of incubation as compared to the growth in the control. The MFC was defined as the lowest concentration of the compound tested without the visual growth of microorganisms [34,35,36]. The MFC/MIC ratio was estimated to investigate the fungicidal (≤4) or fungistatic (>8) effects of the compounds tested. Experiments were repeated in triplicate. Representative data are shown [34,35,36].

## 5. Conclusions

The study revealed that introducing a halogen atom on the phenyl ring of thiosemicarbazides significantly influences the biological activity of the compounds. Fluorine-containing derivatives (**5**–**9**) demonstrated higher activity than their chlorinated counterparts (**2**–**4**). The position of the halogen atom in the phenyl ring proved crucial: the highest activity was observed for derivatives with halogen in the *meta* position (**6**), while substitution in the *para* position was associated with enhanced selectivity of action. Among electron-donating substituents, only the derivative bearing a methoxy group in the *meta* position of the phenyl ring exhibited strong and broad-spectrum activity, whereas relocating this group to the *ortho* or *para* position resulted in a complete loss of activity. Notably, the introduction of the trifluoromethylphenyl moiety, a well-known pharmacophore, led to the disappearance of antifungal properties.

Further derivatives containing both bromine and fluorine atoms could be synthesized to verify whether halogens are indeed responsible for the observed anti-dermatophytical activity. This would establish whether fluorine represents the most favorable halogen for achieving the highest antifungal activity within this class of thiosemicarbazide derivatives.

## Figures and Tables

**Table 1:** Summary of anti-dermatophyte activity of tested thiosemicarbazide compounds against *Trichophyton* spp.

No. of Compound	*T. mentagrophytes* ATCC 9533			*T. mentagrophytes* Clinical Isolate			*T. rubrum* ATCC 28188			*T. rubrum* Clinical Isolate		
	MIC	MFC_50_	MFC_90_	MIC	MFC_50_	MFC_90_	MIC	MFC_50_	MFC_90_	MIC	MFC_50_	MFC_90_
	[µg/mL]											
**1**	250	250	500	125	500	1000	125	125	250	250	250	500
**2**	250	250	250	250	250	500	62.5	62.5	125	62.5	125	250
**3**	125	125	250	250	500	250	62.5	62.5	125	62.5	125	250
**4**	500	500	500	250	500	250	62.5	125	250	125	500	500
**5**	125	125	125	250	250	250	62.5	62.5	125	62.5	125	250
**6**	125	125	125	125	500	250	31.25	62.5	125	62.5	62.5	125
**7**	125	125	125	125	125	250	31.25	125	125	62.5	125	250
**8**	250	250	250	250	500	250	125	125	125	62.5	125	250
**9**	250	1000	2000	1000	500	1000	125	500	1000	250	500	1000
**10**	250	250	500	500	500	500	250	250	250	250	250	250
**11**	125	125	125	125	250	500	31.25	62.5	125	31.25	62.5	125
**12**	250	250	250	250	250	250	125	125	250	125	125	250
**13**	250	250	250	500	500	1000	125	125	250	125	125	125
**14**	2000	>2000	>2000	250	>2000	>2000	2000	>2000	>2000	125	500	1000
fluconazole	100	100	100	50	12.5	25	12.5	50	100	12.5	25	100

Explanatory notes: MIC, MFC_50,_ and MFC_90_ expressed in µg/mL; the MIC values presented are the average of at least three replicates; no standard deviation was observed, and all results equalled their average.

**Table 2:** The structures of compounds investigated against dermatophytes according to [19,20].

Structure	
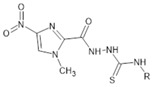	
Compound	R
**1**	C_6_H_5_
**2**	2-ClC_6_H_4_
**3**	3-ClC_6_H_4_
**4**	4-ClC_6_H_4_
**5**	2-FC_6_H_4_
**6**	3-FC_6_H_4_
**7**	4-FC_6_H_4_
**8**	2-CF_3_C_6_H_4_
**9**	3-CF_3_C_6_H_4_
**10**	2-OCH_3_C_6_H_4_
**11**	3-OCH_3_C_6_H_4_
**12**	4-OCH_3_C_6_H_4_
**13**	2-CH_3_C_6_H_4_
**14**	3-CH_3_C_6_H_4_

**Figure A2:**
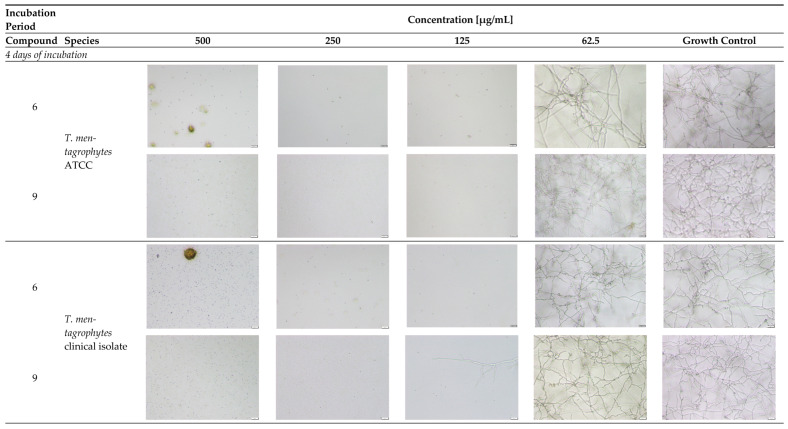

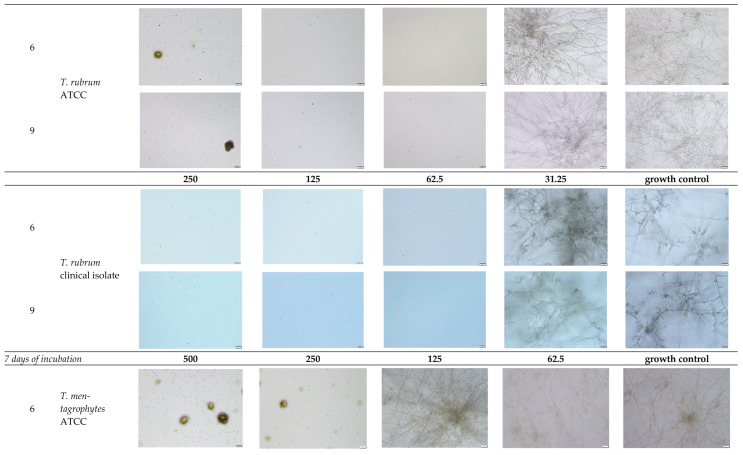

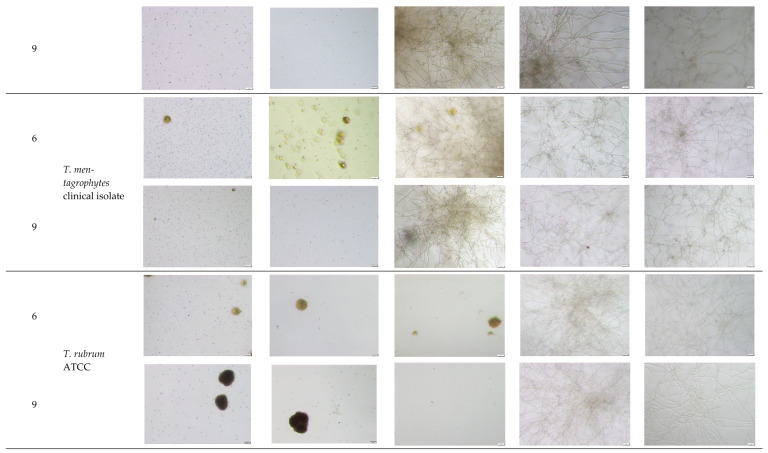

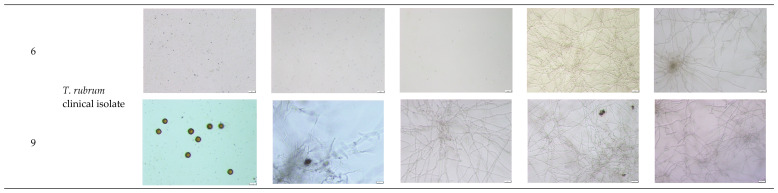
Microscopic observation of 6 and 11 derivatives against dermatophytes studied in relation to the incubation period.

## Data Availability

The original contributions presented in this study are included in the article. Further inquiries can be directed to the corresponding author.

## Abbreviations

The following abbreviations are used in this manuscript:

MIC	minimal inhibitory concentration
MFC	minimal fungicidal concentration
MFC_50_	the lowest concentration that establishes a predetermined reduction of 50% of fungal growth after 5–7 days of incubation, as compared to the growth in the control
MFC_90_	the lowest concentration that establishes a predetermined reduction of 90% of fungal growth after 5–7 days of incubation, as compared to the growth in the control

## Appendix A

Explanatory notes: 2000× magnification, 20 µm scale bar, Olympus DP22 automated inverted light microscope, CellSens Dimensions 2.3 software, Olympus Corporation.
